# Management of Anastomotic Strictures Following Roux-en-Y Hepaticojejunostomy: Our Experience

**DOI:** 10.7759/cureus.105975

**Published:** 2026-03-27

**Authors:** R V Vaishnav Krishna, Rahul Khanna, Ram Niwas Meena, Shashi Prakash, Malayathi Sai Venkatesh

**Affiliations:** 1 General Surgery, Institute of Medical Sciences, Banaras Hindu University, Varanasi, IND

**Keywords:** anastomotic stricture, benign biliary obstruction, bile duct stricture, biliary injury, case series, recurrent biliary stricture, repair cbd injury, roux-en-y complication, roux-en-y hepaticojejunostomy, types of cbd injury

## Abstract

Background: Benign biliary strictures are an uncommon but significant complication following hepatobiliary surgeries, most often seen after cholecystectomy or biliary-enteric anastomosis. While Roux-en-Y hepaticojejunostomy is the standard surgical treatment for high bile duct strictures, recurrence of the stricture remains a complex problem, especially when the anatomy is distorted by fibrosis or previous surgery or when it presents as cholangitis episodes very late after surgery. Revision surgery in these cases is challenging, and timely recognition and resource-appropriate interventions are key to optimal outcomes.

Case summary: We describe a case series of seven patients who underwent revision surgery for recurrent benign biliary strictures following Roux-en-Y hepaticojejunostomy. Three patients were found to have high-level strictures (Strasberg E4) at the time of revision surgery. While most patients were managed with standard surgical techniques, in one of these patients, dense adhesions and scarring made ductal identification nearly impossible, where intraoperative ultrasound-guided biliary catheter placement was used as a salvage technique to localize the biliary ducts transhepatically, creating a neo-hepaticojejunostomy.

Outcome: All patients had a satisfactory recovery, with resolution of jaundice and normalization of liver function over a follow-up period of one year.

Conclusion: Revision surgery for recurrent benign biliary strictures after Roux-en-Y hepaticojejunostomy remains technically demanding due to altered anatomy and fibrosis, where it becomes difficult to differentiate structures. Careful preoperative planning, meticulous intraoperative dissection, flexible surgical strategies, and multidisciplinary cooperation and planning are essential for successful reconstruction and favorable outcomes.

## Introduction

Roux-en-Y hepaticojejunostomy (HJ) is the preferred reconstructive modality of the extra-hepatic bile duct system following iatrogenic injuries, excision of choledochal cyst, and Whipple’s pancreaticoduodenectomy. It is a delicate procedure requiring a high level of surgical expertise and experience. A well-constructed Roux-en-Y HJ gives favorable long-term outcomes; however, the procedure is particularly susceptible to the development of stricture at the anastomotic site.

## Materials and methods

This retrospective observational case series included patients treated in the Department of General Surgery at a tertiary care center in North India (Institute of Medical Sciences, Banaras Hindu University, Varanasi, Uttar Pradesh, India). Patients who underwent revision surgery for recurrent benign biliary strictures following Roux-en-Y HJ between 2015 and 2025 were identified through review of institutional medical records.

Clinical records, operative notes, imaging findings, and postoperative outcomes were reviewed. Data collected included demographic details, indication for the initial surgery, type of biliary injury according to the Strasberg classification, operative findings during revision surgery, and postoperative outcomes. Patients with malignant biliary strictures or incomplete records were excluded. The primary objective of this study was to evaluate the intraoperative surgical challenges and technical strategies/techniques employed during revision surgery. Here, we present our institutional experience in managing these anastomotic strictures occurring following Roux-en-Y HJ.

Cases

A 32-year-old woman, who had undergone an open cholecystectomy for gallstones, developed a bile leak on the first postoperative day. She was referred to our center, where magnetic resonance cholangio-pancreaticography (MRCP) confirmed a Strasberg E1 bile duct injury. After adequate presurgical preparation, she underwent a Roux-en-Y HJ. The postoperative follow-up was uneventful. She presented to us seven months later with progressive jaundice, itching, and elevated bilirubin (total bilirubin = 18.0 mg/dL; direct bilirubin = 12.0 mg/dL; alkaline phosphatase = 454 U/L). MRCP revealed a recurrent high biliary stricture (Strasberg E4), with no communication between the right and left ductal systems. Endoscopic retrograde cholangio-pancreaticography (ERCP) and percutaneous transhepatic stenting failed. She was taken up for redo HJ.

Intraoperatively, dense adhesions were encountered in the subhepatic space and porta hepatis due to previous surgery. Careful adhesiolysis was performed to expose the Roux limb and the hilar region. However, severe fibrosis and inflammation made identification of the right and left hepatic ducts difficult. Attempted mobilization of the Roux limb resulted in detachment of the jejunal limb from the porta hepatis. The right and left hepatic ducts could not be identified visually due to dense scarring at the hilum.

**Figure 1:**
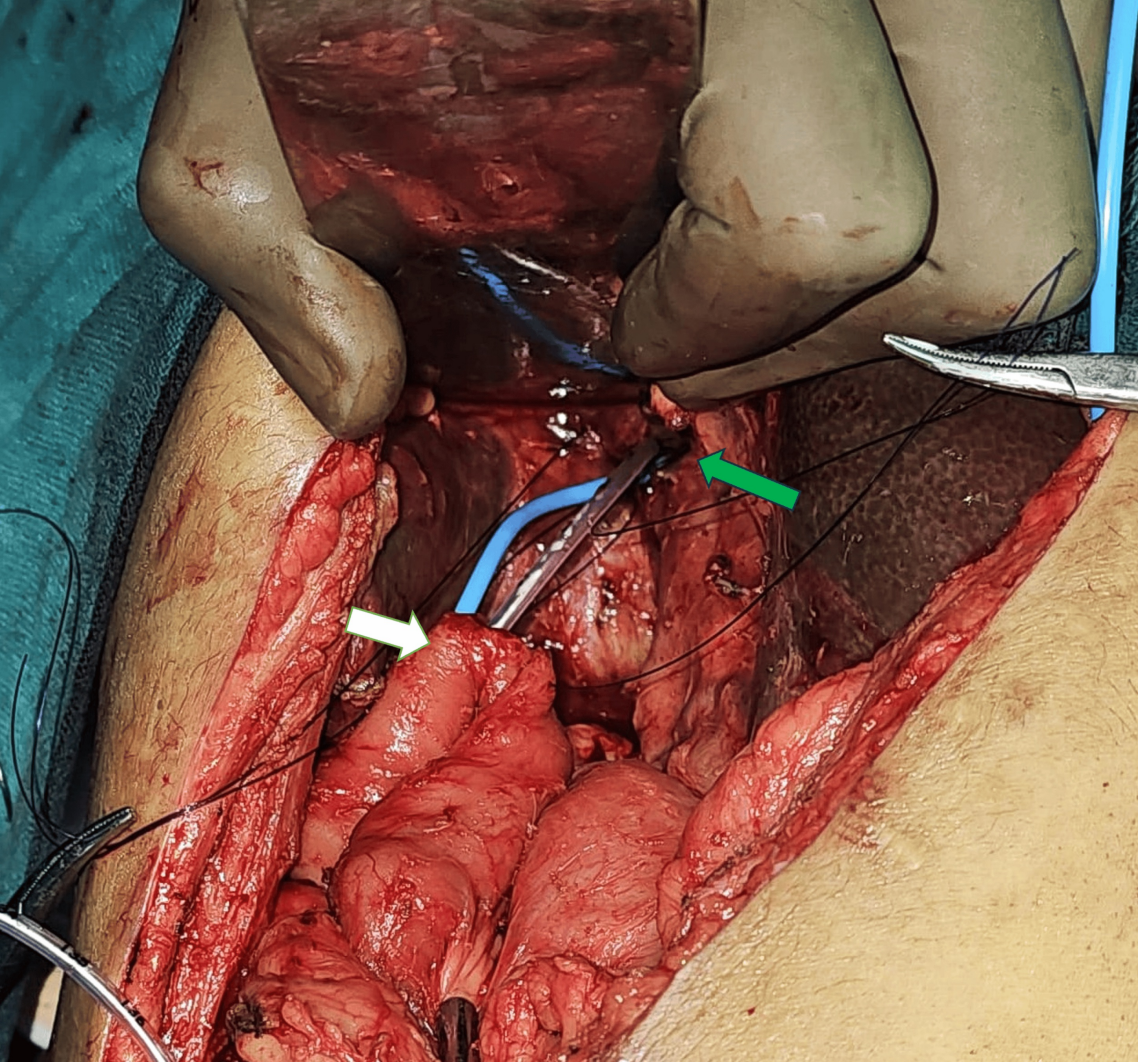
Ultrasound-guided transhepatically placed biliary stent (blue catheter) seen emerging through the left hepatic duct at the porta. Infant feeding tube (white catheter) placed into the right hepatic duct. White arrow: Roux limb; green arrow: porta hepatis at the undersurface of the liver

**Figure 2:**
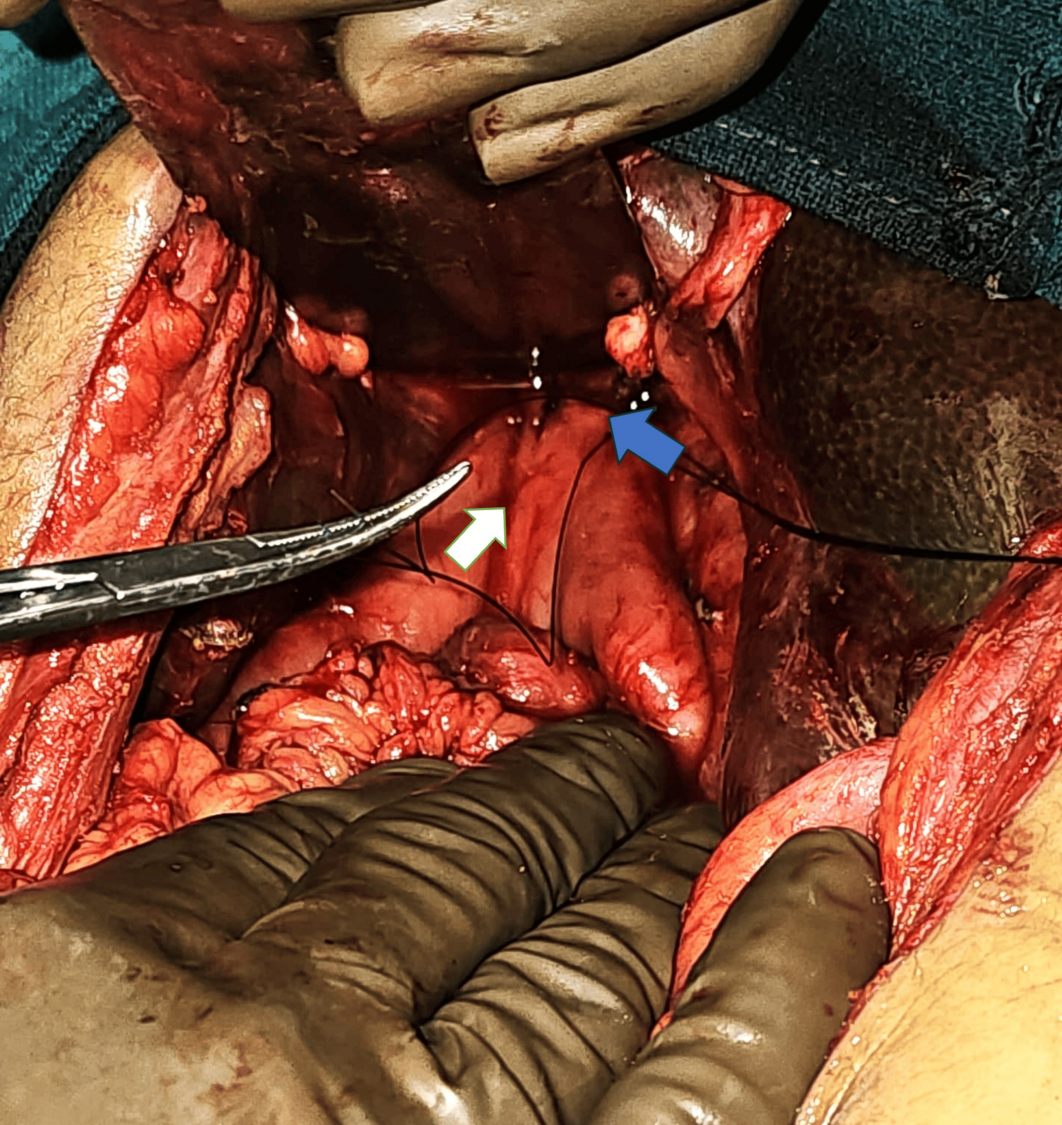
Revision Roux-en-Y hepaticojejunostomy performed over the two tubes described above. White arrow: Roux limb; blue arrow: porta hepatis with neo-hepaticojejunostomy

**Figure 3:**
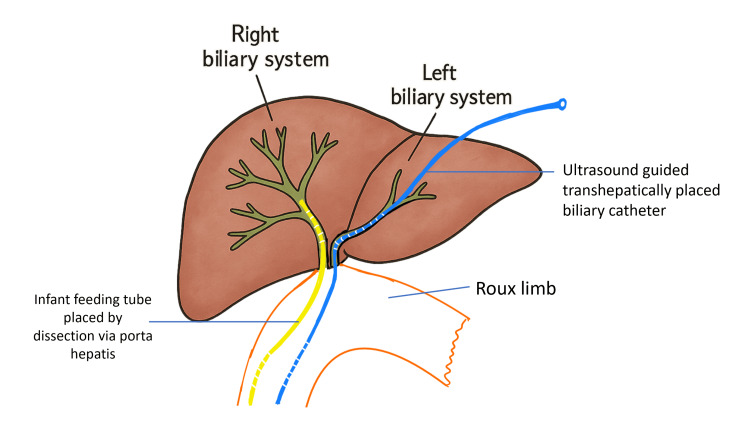
Schematic representation of the procedure done, with the placement of the stents. Original illustration created by the authors using Microsoft PowerPoint (Microsoft Corp., Redmond, WA, USA)

Intraoperative ultrasound was then used to identify the intrahepatic biliary radicals. Under ultrasound guidance, a transhepatic biliary puncture was performed using a needle, and bile was aspirated to confirm entry into the biliary system. A guidewire was passed through the needle into the left hepatic ductal system, and a transhepatic biliary catheter was placed and guided inferiorly toward the porta hepatis. This catheter served as a guide to identify the left hepatic duct. On the right side, the ductal system was identified by careful needle aspiration at the porta hepatis, and an infant feeding tube was inserted into the right hepatic duct to serve as a stent and marker. These catheters helped delineate the biliary anatomy and served as guides for the creation of a neo-HJ (Figures 1, 2). The jejunal Roux limb was then brought up to the hepatic ductal confluence, and a neo-HJ was created by performing a mucosa-to-mucosa anastomosis around the transhepatic catheters using interrupted 4-0 polydioxanone (PDS) sutures. A closed suction drain was placed in the subhepatic space near the anastomosis. Postoperatively, the patient showed gradual clinical improvement. Drain output decreased progressively, and a postoperative cholangiogram performed on postoperative day 10 showed no evidence of bile leak. The transhepatic catheters were removed after two weeks, following confirmation of free flow of contrast into the jejunal limb. A schematic representation of the procedure done is shown in Figure 3.

**Figure 4:**
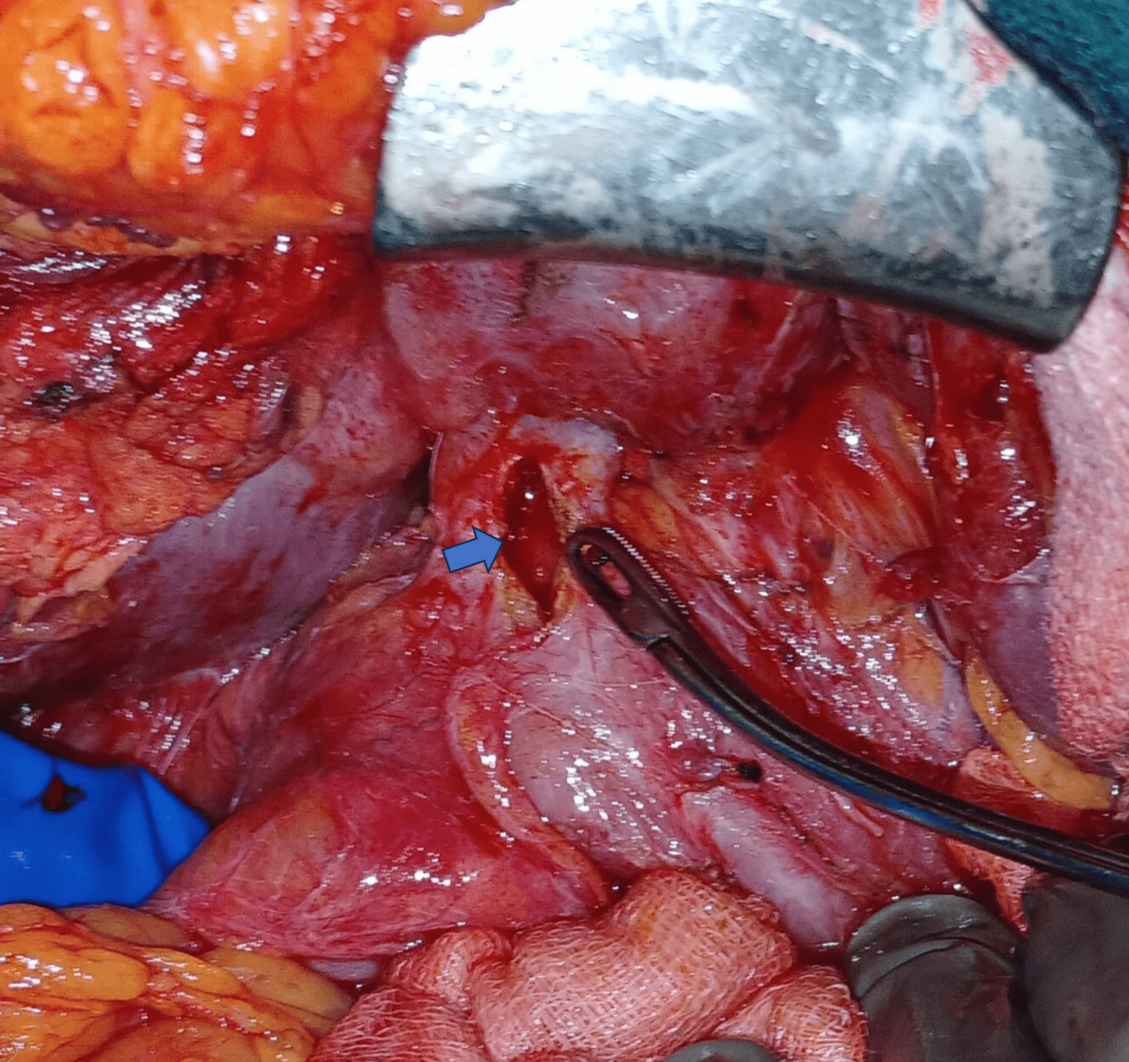
Anastomotic stricture of a previous Roux-en-Y hepaticojejunostomy opened longitudinally. Blue arrow: stricture site in the common hepatic duct that has been longitudinally incised

**Figure 5:**
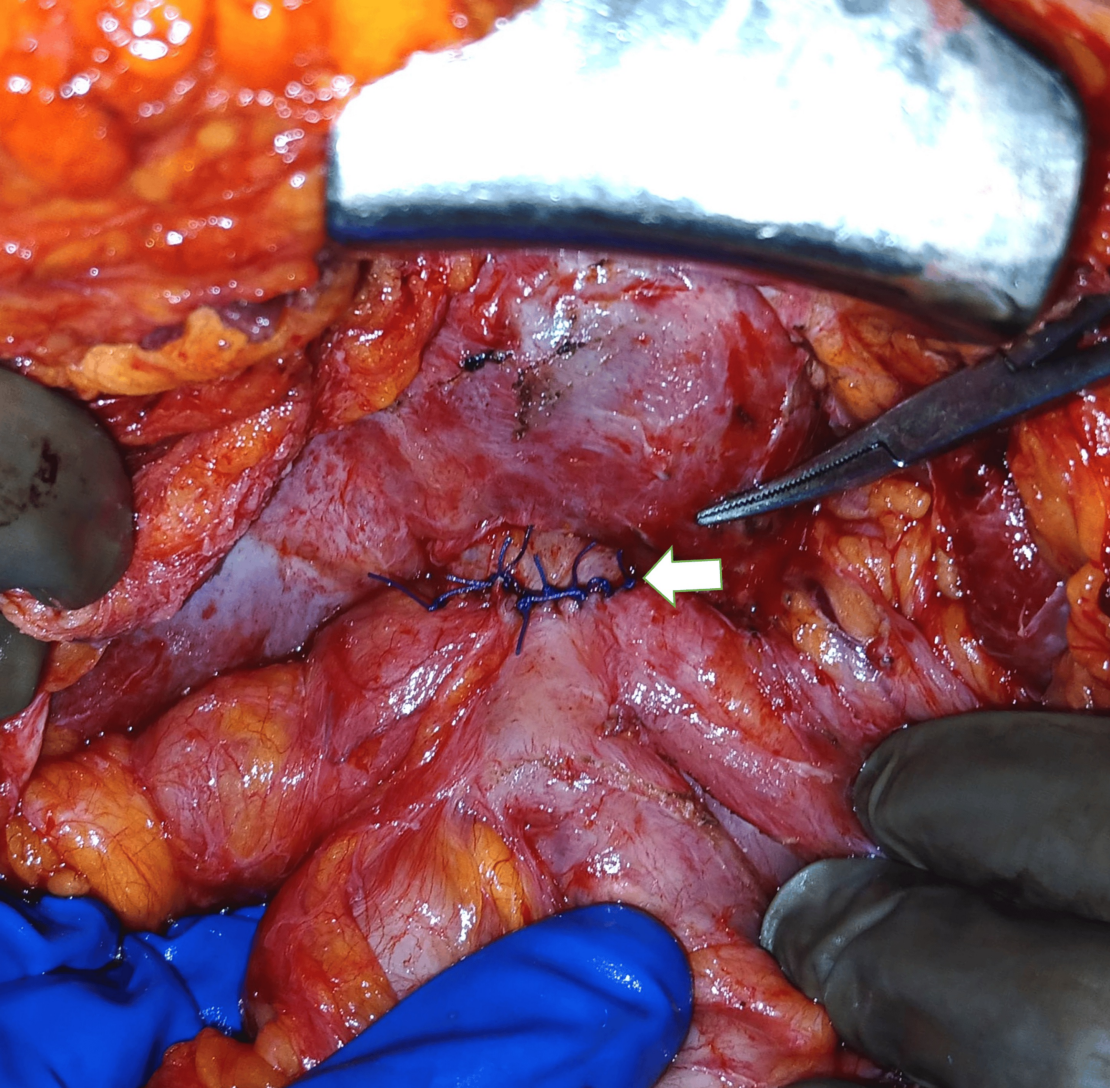
Stricturoplasty accomplished by transverse closure (white arrow) of the longitudinal incision across the anastomotic stricture at the common hepatic duct.

A 66-year-old woman presented with progressive jaundice three years after undergoing Whipple’s procedure with Roux-en-Y HJ for periampullary carcinoma. MRCP demonstrated a stricture at the HJ anastomotic site. At surgery, dense adhesions were encountered in the upper abdomen, which were carefully lysed to expose the Roux limb and the HJ site. The Roux limb was identified and traced to the HJ. The anastomotic site was found to be narrowed, and the proximal bile duct was dilated with an impacted bile duct stone proximal to the stricture. The anastomosis was opened longitudinally over the stricture site, and the bile duct stone was removed. The stricture segment was then incised longitudinally (Figure 4) to include both the bile duct and the jejunal wall at the anastomotic site. The incision was extended until healthy mucosa was visualized proximally and distally to ensure adequate lumen. The stricturoplasty was then performed using the Heineke-Mikulicz principle by closing the longitudinal incision in a transverse fashion (Figure 5) to widen the anastomotic lumen. Interrupted 4-0 PDS sutures were used to approximate the mucosa-to-mucosa edges, ensuring a tension-free anastomosis. Care was taken to achieve precise mucosal approximation to prevent restenosis. The anastomosis was checked for bile flow and hemostasis. A closed suction drain was placed near the anastomotic site. She had an uneventful recovery, with normalization of liver enzymes.

Other cases

A 54-year-old woman, five years post-choledochal cyst excision with Roux-en-Y HJ, came with recurrent cholangitis and abnormal liver tests. MRCP showed a stricture and a stone near the anastomosis. At surgery, the stone was extracted, and stricturoplasty was performed. She improved clinically, with no recurrence of jaundice on follow-up. Four patients with prior Roux-en-Y HJ for post-cholecystectomy biliary strictures presented with jaundice and abnormal liver tests. Imaging confirmed anastomotic strictures. Revision surgery involved excising the fibrotic anastomotic segment and constructing a new HJ at a higher level over identifiable proximal ducts. All four recovered well, with resolution of symptoms and improvement in lab parameters. Follow-up of all the patients was done for at least a year after surgery with liver function tests (LFTs) and clinical examination. The demographic profile of these patients is shown in Table 1.

**Table 1:** Demographic and surgical details of the patients (n = 7). HJ: hepaticojejunostomy; F: female

S. No.	Age/gender	Diagnosis	Index surgery	Initial Bismuth Strasberg grade	Time interval between index surgery and revision surgery	Revision surgery done	Bismuth Strasberg grade at revision Roux-en-Y HJ
1	32/F	Post-cholecystectomy stricture	Roux-en-Y HJ	E1	7 months	Revision Roux-en-Y HJ with the following image-guided biliary catheter placement	E4
2	66/F	Periampullary carcinoma	Roux-en-Y HJ as part of Whipple’s operation	-	18 months	Proximal calculus extraction + stricturoplasty	E3
3	54/F	Choledochal cyst	Excision + Roux-en-Y HJ	-	14 months	Proximal calculus extraction + stricturoplasty	E3
4	51/F	Post-cholecystectomy stricture	Roux-en-Y HJ	E3	10 months	Revision Roux-en-Y HJ	E4
5	24/F	Post-cholecystectomy stricture	Roux-en-Y HJ	E3	11 months	Revision Roux-en-Y HJ	E3
6	71/F	Post-cholecystectomy stricture	Roux-en-Y HJ	E2	12 months	Revision Roux-en-Y HJ	E4
7	34/F	Post-cholecystectomy stricture	Roux-en-Y HJ	E2	8 months	Revision Roux-en-Y HJ	E3

Postoperatively, patients were followed up clinically and biochemically. LFTs were performed at regular intervals (one month, three months, six months, and 12 months). Imaging in the form of abdominal ultrasound was performed during follow-up, and MRCP was performed in patients with symptoms suggestive of stricture recurrence. Follow-up data were obtained from outpatient records. The minimum follow-up duration was 12 months.

## Results

Over a period of 10 years, from 2015 to 2025, we performed revision surgery on seven patients who developed anastomotic stricture following Roux-en-Y HJ. Of these, five had undergone Roux-en-Y HJ for a post-cholecystectomy stricture; one had Roux-en-Y HJ as part of Whipple's pancreaticoduodenectomy, and one had Roux-en-Y HJ following excision of a choledochal cyst. In the five patients who had undergone the initial Roux-en-Y HJ following a post-cholecystectomy stricture, the stricture was categorized as per Bismuth Strasberg grading system as follows: Bismuth Type E1:1, Bismuth Type E2:2, and Bismuth Type E3:2. The patient who had undergone Whipple's operation and choledochal cyst excision did not have a biliary stricture at the time of the first operation, and a 2-3 cm proximal common hepatic duct (CHD) stump was available at revision surgery in these cases (Table 1).

At the time of revision surgery, the stricture was found to have migrated proximally in most of the patients (four patients) who had undergone Roux-en-Y HJ for iatrogenic bile duct stricture, as documented in Table 1. In four patients (including Whipple's and choledochal cyst excision), the stricture was found at the confluence, while in three patients (post-cholecystectomy cases), the stricture had migrated proximal to the confluence with separation of the right and left hepatic ducts (Bismuth Strasberg Grade E4). Intraoperative identification of the proximal bile duct at the time of revision surgery was possible in six patients with meticulous surgical dissection.

A hepatic duct calculus was found in two patients (Whipple's and choledochal cyst cases), and both were managed with stone extraction and a stricturoplasty of the anastomotic site. The presence of hepatic duct calculi in these patients was likely secondary to biliary stasis resulting from partial narrowing at the anastomotic site. In these cases, the anastomosis remained identifiable, and the proximal ductal anatomy was preserved; therefore, stone extraction followed by stricturoplasty was considered adequate, avoiding the need for a redo HJ. Among the five patients with a post-cholecystectomy stricture, four required a revision Roux-en-Y HJ. In one patient (32-year woman) with a Bismuth Strasberg Grade E4 stricture, dissection of liver parenchyma at the porta hepatis was required. The index surgery was a cholecystectomy complicated by a Strasberg E1 injury, managed with a Roux-en-Y HJ. No major resection of the hepatic duct was documented. At the time of revision surgery, the stricture had progressed to a Strasberg E4 level, and intraoperative ultrasound-guided transhepatic stent placement in the left hepatic duct was done.

All the patients had an uneventful postoperative recovery. LFTs showed normalization or significant improvement. During the follow-up period of one year, patients were monitored with clinical examination, serial LFTs, and abdominal ultrasound. None of the patients developed cholangitis or radiological evidence of anastomotic stricture recurrence during the follow-up period. No patient required re-intervention.

## Discussion

Benign biliary strictures most commonly occur as a complication of iatrogenic bile duct injury, with cholecystectomy accounting for the majority of cases, particularly in low-resource and emergency settings. While Roux-en-Y HJ remains the gold standard for definitive surgical management of high biliary strictures, recurrent benign biliary strictures following Roux-en-Y HJ remain a formidable surgical challenge, despite advances in imaging, surgical techniques, and perioperative care. Reported recurrence rates range between 10% and 30%, with most cases attributed to ischemia, anastomotic tension, lack of duct dilation, fibrotic healing, or unrecognized proximal injury during the index surgery [1-3]. Management of such strictures requires technical expertise, individualized strategies, and a multidisciplinary approach based on the stricture's level, extent, and underlying etiology.

Observations across the series

Several patterns emerged in our 10-year experience, comprising seven patients undergoing revision surgery for recurrent biliary strictures after Roux-en-Y HJ. Five patients had undergone HJ for post-cholecystectomy strictures, while two had undergone HJ as part of complex procedures (Whipple's operation and choledochal cyst excision). Among patients with post-cholecystectomy biliary injuries, the level of stricture at revision surgery was frequently (four patients) found to be more proximal than that documented at the index procedure, an observation consistent with reports by Halle-Smith et al. and Yan et al., where progressive fibrosis led to the involvement of the biliary confluence and proximal hepatic ducts over time [4,5].

**Figure 6:**
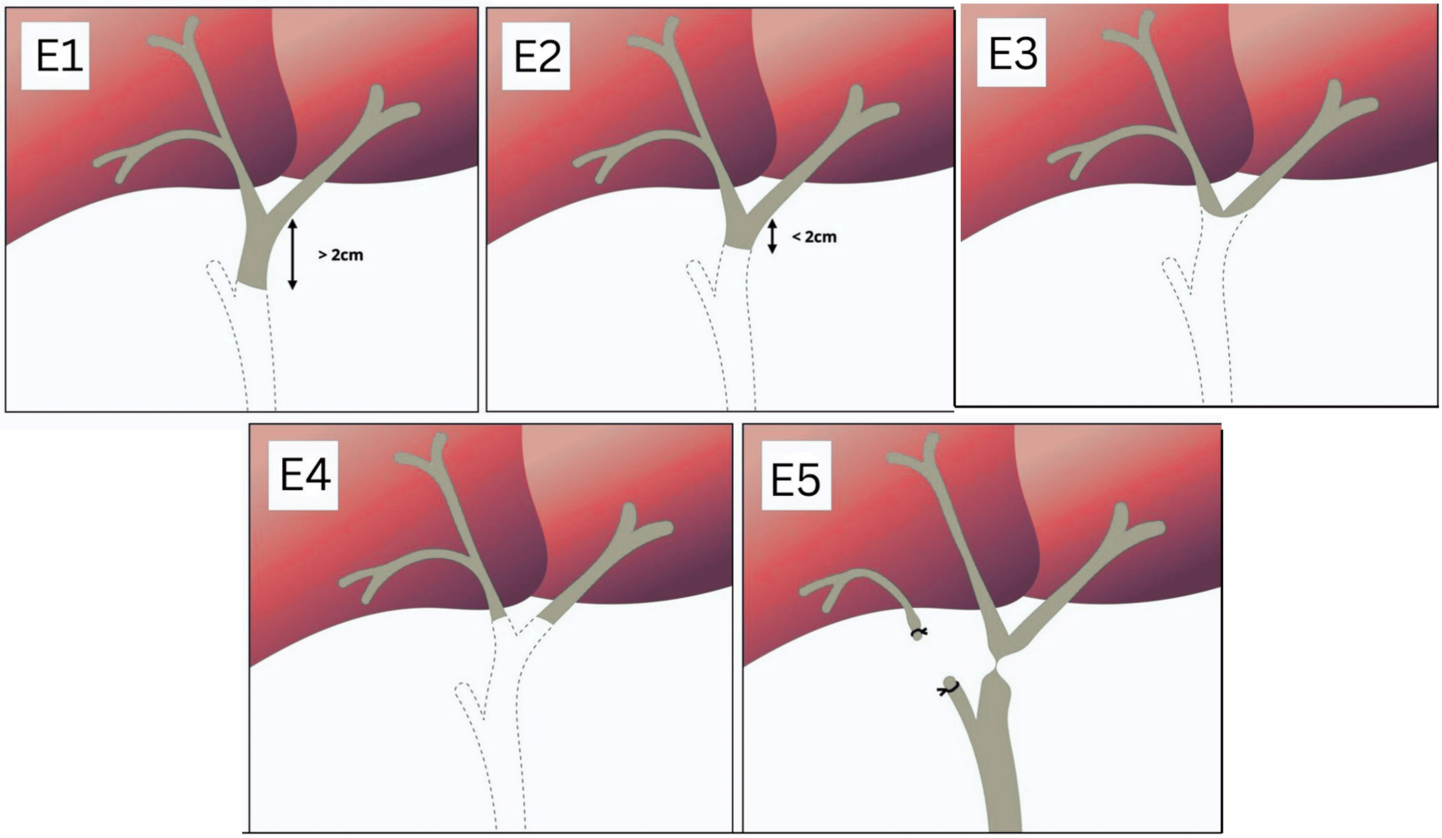
Strasberg classification of bile duct injuries. Original illustration created by the authors using Microsoft PowerPoint based on information from Radiopaedia.org [7] (Creative Commons Attribution-NonCommercial-ShareAlike 3.0 Unported (CC BY-NC-SA 3.0))

At revision surgery, four patients had strictures at the biliary confluence (Strasberg E3), while three had migrated proximal to the confluence, separating the right and left hepatic ducts (Strasberg E4). This proximal migration increases surgical complexity by reducing available extra-hepatic duct length for anastomosis and necessitating higher-level dissection [6]. The Strasberg classification of bile duct injuries is the most commonly used classification system for the same and is shown in Figure 6.

Two patients developed intraductal calculi proximal to the anastomosis, likely due to bile stasis at the stricture site - a known complication reported in 15%-20% of post-HJ strictures [8]. Both were successfully managed with stone extraction and stricturoplasty, aligning with evidence that stricturoplasty may be considered in select cases with short-segment fibrosis and good tissue quality [9].

Technical considerations and surgical expertise needed in revision surgery

Revision HJ poses substantially greater technical difficulty compared to primary HJ, due to dense adhesions, distorted anatomy, and obliteration of tissue planes [10]. This was encountered in all patients in our series. The literature emphasizes meticulous adhesiolysis, early identification of vasculobiliary landmarks, and avoidance of excessive traction to prevent further vascular injury [11]. There are several factors that can predispose a Roux-en-Y HJ to stricture formation. They are the presence of an associated biliary fistula, non-dilated proximal hepatic duct, a high anastomosis at confluence or proximal to it, excessive use of electro-cautery, revision surgery, and combined bilio-vascular injury. In our experience, we found one or a combination of more than one of the above factors in all seven cases in which a revision Roux-en-Y HJ was done. The biggest challenge in revision surgery was the dissection of the fibrotic adhesions for delineating the strictured Roux-en-Y without injury to the duodenum, transverse colon, portal vein, or hepatic artery. We used electro-cautery judiciously at a low power setting.

There are three technical challenges that confront a surgeon undertaking revision surgery for a strictured Roux-en-Y HJ. These are the lysis of the dense adhesions to visualize the porta hepatis, identification of the hepatic duct and left and right hepatic duct stumps, and preservation of good vascularity of the stumps. The basic requirements of a tension-free anastomosis of two well-vascularized structures of adequate luminal size have to be met for a successful outcome.

The proximal bile duct could be identified in six of seven patients through careful dissection. However, in one patient with a Strasberg E4 stricture, neither the right nor left hepatic ducts were identifiable visually despite extensive dissection and needle aspiration attempts (to be done with great caution due to potential risks involved) at the porta hepatis. While progressive fibrosis and ischemic changes are the primary culprits for this proximal migration, other possible factors include recurrent inflammation, devascularization during the initial dissection, and secondary traction at the anastomotic site. These mechanisms may lead to progressive proximal extension of strictures, a finding consistent with published reports of benign biliary stricture evolution. Preoperative percutaneous transhepatic cholangiography (PTC) was attempted in this case to access the proximal biliary system; however, it was unsuccessful due to the high level of obstruction and distorted biliary anatomy. Consequently, intraoperative image-guided (ultrasound) transhepatic biliary catheter placement was employed not as a primary modality but as a rescue technique/bailout procedure, aiding safe localization of the right and left ductal systems transhepatically, by advancing the catheter under image guidance, which ultimately helped in identifying the left hepatic duct, which was now found to be intrahepatic, in the absence of external identifiable structures. This allowed successful reconstruction when conventional dissection was no longer feasible. This approach resembles reports by Hakamada et al. and Chandra et al., where intraoperative ultrasonography or fluoroscopy facilitated transhepatic ductal access when standard dissection failed [12,13].

Though intraoperative ultrasound is widely used in hepatic resections and transplantation, its application in benign biliary reconstruction is rarely described in the literature. Similar principles of guided ductal access and neo-anastomosis have been described only in select transplant and trauma cases [14]. Recent advances such as near-infrared fluorescence cholangiography (NIFC), indocyanine green (ICG)-based imaging, and intraoperative 3D navigation have shown promise in delineating biliary anatomy in hepatobiliary surgery, though their application in redo HJ is still limited to case reports and feasibility studies [15,16]. Wider adoption of these adjuncts may further reduce morbidity in complex revisions. Our experience adds to this limited body of evidence by demonstrating its feasibility as a real-time adjunct for ductal localization in complex reoperative settings.

When to do revision HJ or stricturoplasty

While revision HJ remains the standard treatment for long or high strictures, stricturoplasty offers a useful alternative in selected cases. Revision HJ requires extensive hilar dissection and may necessitate a higher anastomosis, increasing technical difficulty and risk of vascular injury. In contrast, stricturoplasty is less extensive, preserves the existing anastomosis, and may reduce operative time and morbidity when performed in appropriate cases. Five patients underwent revision Roux-en-Y HJ in our series, while two were managed with stricturoplasty. The choice depended on the degree of fibrosis, stricture length, presence of ductal stones, and intraoperative assessment of ductal tissue quality. While stricturoplasty preserves ductal length and avoids a higher anastomosis, its role is limited to short, non-ischemic strictures without significant proximal fibrosis [9,17]. The risk of stricture recurrence increases with proximal anastomosis, anastomotic ischemia, or technical failure during revision. For these reasons, some centers advocate stenting or staged reconstruction in select high-risk patients [18,19]. Longer or proximal strictures, particularly at or above the confluence, necessitate revision HJ at a higher level to ensure drainage of both the right and left systems. Similar findings have been reported in large reoperative series by de Santibáñes et al. and Moris et al., who emphasized individualized surgical planning based on intraoperative findings [2,18]. Although our patients did not undergo stenting, some centers advocate temporary transanastomotic stenting or internal-external drainage in high-risk revisions to reduce early stricture recurrence, though data remain conflicting [18,20]. Our series highlights that not all recurrent HJ strictures require revision anastomosis; in selected cases with short-segment fibrosis and favorable ductal tissue, stricturoplasty can provide satisfactory outcomes while avoiding morbidity associated with redo HJ.

Outcomes of our cases

All patients in our series had uneventful postoperative recovery with normalization of liver function and resolution of symptoms. They were followed up with serial LFTs, clinical examination, and abdominal ultrasound for a period of one year. This favorable outcome aligns with published reports showing 80%-90% long-term patency rates after revision HJ, though close surveillance remains critical [3,4].

Limitations of the study

This study has several limitations. First, the sample size is small (n = 7) due to the rarity and complexity of recurrent benign biliary strictures requiring revision HJ. Second, this is a retrospective observational study and is therefore subject to inherent selection and information bias. Third, this study represents the experience of a single tertiary care center, which may limit generalizability. Despite these limitations, the study highlights important intraoperative challenges and demonstrates the feasibility of intraoperative ultrasound-guided biliary catheter placement in complex biliary strictures.

Lessons learned and our recommendations

Our experience reinforces several key lessons in managing recurrent benign biliary strictures: preoperative imaging (MRCP, PTC) must be interpreted in conjunction with intraoperative findings, recognizing potential proximal migration of strictures. Meticulous intraoperative dissection is paramount as standard landmarks may be absent or distorted. Therefore, intraoperative image-guided techniques (ultrasound, cholangiography) can be valuable adjuncts in ductal localization, especially in complex reoperative fields, when the extra-hepatic ductal system cannot be identified at the porta hepatis by dissection. Stricturoplasty may be considered selectively in short-segment strictures with favorable tissue; otherwise, revision HJ remains the standard. An individualized, multidisciplinary approach enhances intraoperative decision-making and improves outcomes.

## Conclusions

Revision HJ for recurrent benign biliary strictures remains a demanding surgical procedure. While meticulous dissection and anatomic reconstruction are very demanding and of paramount importance, adjunctive intraoperative imaging may play a critical role in selected cases where anatomy is obscured. Our case series adds to the growing ocean of evidence supporting individualized surgical strategies tailored to intraoperative findings, reinforcing that successful outcomes are achievable even in the most complex scenarios with careful planning, adaptability, and technical precision.
